# Sperm enrichment from poor semen samples by double density gradient centrifugation in combination with swim-up for IVF cycles

**DOI:** 10.1038/s41598-020-59347-y

**Published:** 2020-02-10

**Authors:** Xiuliang Dai, Yufeng Wang, Fang Cao, Chunmei Yu, Tingting Gao, Xiyang Xia, Jun Wu, Li Chen

**Affiliations:** 1 Department of Reproductive Medicine Center, the Affiliated Changzhou Maternal and Child Health Care Hospital of Nanjing Medical University, Changzhou, Jiangsu 213000 P.R. China; 2 The Research Center for Bone and Stem Cells, Department of Anatomy, Histology and Embryology, Nanjing Medical University, Nanjing, 210029 China

**Keywords:** Infertility, Adverse effects

## Abstract

Sperm preparation in IVF cycles using density gradient centrifugation (DGC) in combination with swim-up (SU) has been widely adopted in reproductive centres worldwide. It is a fact that the sperm recovery rate following one DGC from poor semen samples (showing liquefaction defects/containing too many unresolvable clots or rare sperm) is relatively low. Our results showed that double DGC (DDGC) is effective at increasing the sperm recovery rate from poor semen samples. However, DDGC may increase the mechanical stress of sperm, thereby potentially impairing embryo development. Therefore, it is necessary to evaluate the safety of using sperm prepared by DDGC/SU for IVF cycles. In this study, we retrospectively analysed the data generated from a total of 529 IVF cycles (from June 2017 to June 2018), and these IVF cycles contributed 622 transfer cycles (from June 2017 to December 2018) in Changzhou Maternal and Child Health Care Hospital. Of them, 306 IVF cycles and the related 355 transfer cycles (normal semen samples prepared by DGC/SU) were set as the normal group, while 223 IVF cycles and the related 267 transfer cycles (poor semen prepared by DDGC/SU) were set as the observation group. The main outcome measures, including the normal fertilization rate, top D3 embryo formation rate, blastocyte formation rate, clinical pregnancy rate and live birth rate, birth weight and duration of pregnancy, were compared between the two groups. Compared to semen in the DGC/SU group, semen in the DDGC/SU group showed increased levels of the DNA fragmentation index (DFI) and reduced sperm concentration, percentage of progressive motility (PR) sperm, and percentage of normal morphology sperm. The indicators reflecting *in vitro* embryo development and clinical outcomes were similar in the DGC/SU group and DDGC/SU group, including the normal fertilization rate, top D3 embryo formation rate, blastocyte formation rate, pregnancy rate, implantation rate, spontaneous abortion rate, live birth rate, birth weight and duration of pregnancy. Furthermore, we found that the 1PN zygote formation rate was significantly lower in the DDGC/SU group than that in the DGC/SU group. We concluded that oocytes fertilized by sperm from poor semen samples separated by DDGC/SU achieved the same outcomes as oocytes fertilized by sperm from normal semen separated by DGC/SU, suggesting that DDGC/SU is an effective and safe method of sperm enrichment for poor semen samples in IVF. The main contribution of the present study is the verification of the effectiveness of DDGC/SU in improving sperm recovery from poor semen samples and the safety of using sperm prepared by DDGC/SU for IVF.

## Introduction

In *in vitro* fertilization (IVF), two major semen preparation methods have been widely used for selecting sperm from semen, namely density gradient centrifugation (DGC) and swim-up (SU). Numerous studies have focused on investigating which method is better for sperm enrichment. However, studies from different groups have drawn inconsistent conclusions^1–6^. Recent studies have suggested that DGC in combination with SU (DGC/SU) could be one of the most effective approaches^7,8^. Most Chinese reproductive centres, including ours, have adopted DGC/SU for enriching sperm in IVF.

In clinical sperm preparation, the sperm recovery rate following one DGC largely depends on semen quality. Generally speaking, the sperm recovery rate from poor semen samples (such as liquefaction defects, containing too many unresolvable clots or rare sperm) is far from satisfactory. In clinical settings, when purifying sperm from semen for conventional IVF, we often encounter the situation where only a small proportion of sperm from a poor semen sample was obtained after one DGC. We have to make a choice: ignore the remaining motile sperm in the semen or re-extract them. In our reproductive centre, if few sperm are obtained after one DGC, the sperm precipitation (visible or invisible) is collected, and the remaining semen undergoes a second round of DGC with the old gradient column. Actually, the sperm from the double consecutive DGC would be pooled together for the next swim-up procedure. Here, we defined the double consecutive DGC in combination with SU as DDGC/SU.

It has been demonstrated that an increase in the centrifugation time or gravitational (g) force can increase the sperm recovery rate from equine semen but can also decrease sperm motility or quality due to the mechanical forces associated with centrifugation and excessive packing of the sperm^9^. Studies using spermatozoa from the epididymis of rats and mice revealed that increasing the centrifugation force decreased sperm function^10–12^. A previous study demonstrated that centrifugation stress reduces the responsiveness of the spermatozoa of pigs to a capacitation stimulus^13^. Although the sperm of bull are relatively resistant to high centrifugal forces, it has been demonstrated that a reduction in centrifugation force has increased the fertilization rate *in vitro*^14^. Studies investigating the influence of centrifugation on human sperm showed that centrifugation induced damage to human sperm^15,16^. Indeed, in the abovementioned studies, either conventional centrifugation or DGC was used for sperm preparation. Although it is believed that DGC causes less damage to sperm than conventional centrifugation, centrifugation per se is harmful^16^. Therefore, it raises the concern that mechanical stress induced by either the increased centrifugation force or additional centrifugation time will further increase sperm damage, which may potentially affect the process of fertilization or embryo development. However, studies correlating the mechanical stress of sperm with embryo development are lacking. Although it has been demonstrated that sperm processing by DGC and SU is effective at reducing sperm with DNA damage, which is thought to definitely adversely affect early embryo development, it remains elusive whether a second DGC will introduce new damage to sperm, thereby finally impairing embryo development^17,18^.

Although the sperm from both the first and second DGC were gathered, the safety of DDGC/SU for sperm needs to be evaluated. In this study, we retrospectively analysed the data generated from patients from June 2017 to December 2018 in the reproductive centre of Changzhou Maternal and Child Health Care Hospital for IVF treatment. According to the method of sperm processing, patients were divided into two groups: the DGC/SU group and the DDGC/SU group. The data generated in this study were compared between the two groups.

## Results

### A second DGC significantly increased the motile sperm recovery rate from poor semen samples

**Figure 1:**
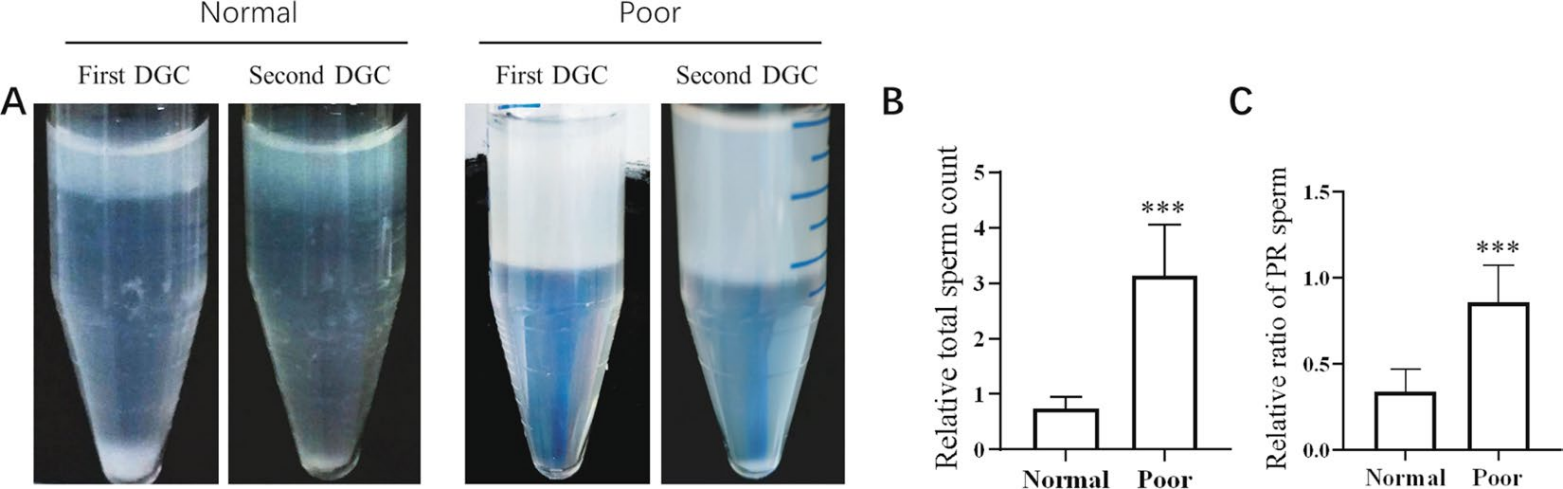
A second DGC significantly improves the recovery rate of motile sperm from poor semen samples. Normal or poor semen samples underwent two consecutive DGCs (462 g for 15 min), (**A**) Representative images of sperm precipitation after the first DGC (left tube) and second DGC (right tube). (**B**) Ratio of the total sperm count in the second DGC to the first DGC. (**C**) Ratio of PR sperm in the second DGC to first DGC. Normal semen samples vs. poor semen samples; ***P < 0.001.

To determine whether a second DGC will increase the recovered sperm from semen, we separated and collected the sperms from normal and poor semen samples by first and second DGCs, and the parameters of sperm from the first and second DGCs were analysed. The results showed that an amount of sperm could still be extracted from normal or poor semen samples by the second DGC. However, the relative total sperm count and the ratio of PR-sperm acquired by the second DGC to the first DGC were much higher from poor semen samples than those from normal semen samples (Fig. 1A–C). These results indicated that the second DGC effectively separated motile sperm from poor semen samples.

### Basic characteristics of patients

The present study included a total of 529 IVF cycles, 306 cycles for the DGC/SU group and 223 cycles for DDGC/SU. The patient characteristics and cycle parameters were compared between the two groups as shown in Table 1.The percentage of primary infertility, infertility duration, paternal or maternal age and BMI, ovulation induction protocols, basal hormonal levels, total gonadotropin (Gn) use, duration of Gn use, endometrial thickness, average oocytes retrieved and MII oocytes were comparable in the DGC/SU and DDGC/SU groups (Table 1). The DNA fragmentation index (DFI) was significant higher in the DDGC/SU group than that in the DGC/SU group, while the sperm concentration, the proportion of progressive motility (PR) and normal morphology sperm were lower in the DDGC/SU group (Table 1). In addition, the proportion of male-infertility factors was significantly higher in the DDGC/SU group (Table 1).

**Table 1:** Patient characteristics and cycle parameters.

	DGC/SU	DDGC/SU	p value
IVF cycles	306	223	
Primary infertility (%)	150 (49.0)	125 (56.0)	0.1098
Infertility duration(years)	3 [2,4]	3 [2,5]	0.6637
Age			
Male	31 [28,35]	31 [29,36]	0.2888
Female	30[28,34]	31[28,34]	0.1716
BMI			
Male	24.6 [22.5,27.0]	24.2 [22.7,27.2]	0.8179
Female	21.80 [19.9,24.6]	22.2 [20.3,24.8]	0.1638
Parameter of semen quality*			
Concentration (10^6^/ml)	63.2[37.3,97]	46.3 [28.2,72.9]	0.0001
Total sperm count (10^6^)	172.2 [105.7,279.1]	132 [85.1,242.9]	0.0038
PR (%)	47.14 ± 17.49	35.48 ± 17.09	<0.0001
Sperm DFI	10 [6.9,14.6]	13.0 [8.6,19.4]	<0.0001
Normal morphology (%)	4.3 [4.1,4.8]	4.1[3.7,4.4]	<0.0001
Diagnosis			
Tubal (%)	153 (50)	99 (44.4)	0.2024
Endometriosis (%)	17 (5.6)	10 (4.5)	0.5804
Declined ovarian reserve (%)	31 (10.1)	28 (12.6)	0.3815
Male factor (%)	10 (3.3)	25 (11.2)	0.0003
Female hormonal level			
Basal FSH, IU/l	6.6 [5.5,7.9]	6.4 [5.3,7.6]	0.1218
Basal LH, IU/l	4.9 [3.4,7.0]	4.7 [3.5,6.5]	0.4462
Base E2, ng/l	29.8 [18.3,43.9]	31.1 [18.3,44.8]	0.7778
AMH, ng/ml	2.9 [1.5,5.3]	3.1 [1.6,5.5]	0.4628
GnRH analogues			
Agonist (%)	153 (50)	116 (52)	0.6466
Antagonist (%)	56 (18.3)	43 (19.3)	0.7749
No analogues (%)	97 (31.7)	64 (28.7)	0.5033
Total dose of Gn	2025 [1316,2700]	1950 [1347,2700]	0.7650
Duration of Gn treatment (days)	9 [8,10]	9 [8,10]	0.7816
Endometrial thickness	8.0 [9.0,10.0]	8.0 [9.0,11.0]	0.8737
Average oocytes retrieved	8 [5,12]	9 [5,13]	0.2391
Average MII oocytes	8 [5,11]	8 [5,13]	0.3899

DFI, DNA fragmentation index; Gn, Gonadotropin. Data are presented as the median [the first quartile, the third quartile] or count (percentage). *Semen was ejaculated for CASA analysis in andrology clinic 2 weeks before insemination.

### Comparison of *in vitro* embryo developmental parameters between the DGC/SU and DDGC/SU groups

The results showed that the rate of normal fertilization and the formation rate of top D3 embryos (grade I and II) from either 2PN or 1PN zygotes and blastocysts from either top or not top D3 embryos (grade III) were comparable in the DGC/SU group and DDGC/SU group (Table 2). However, the 1PN zygote formation rate was significantly lower in DDGC/SU group than that in the DGC/SU group (Table 2).

**Table 2:** *In vitro* embryo developmental parameters.

	DGC/SU	DDGC/SU	p value
Normal fertilization (%)	1957/2478 (79.0)	1569/1978 (79.3)	0.3928
1PN/MII oocytes (%)	124/2478 (5.0)	63/1978 (3.2)	0.0026
Top D3 embryos from 2PN (%)	1387/1957 (70.9)	1146/1569 (73.0)	0.6156
Top D3 embryos from 1PN (%)	36/124 (29.0)	17/63 (27.0)	0.7690
Blastocysts from top D3 embryos (%)	469/729 (64.3)	431/631 (68.3)	0.1228
Blastocysts from non-top D3 embryos (%)	67/347 (19.3)	54/220 (24.5)	0.1380

PN: pronucleus. Data are presented as counts (percentages). Top D3 embryos refers to the grade1 and grade2 embryos. Non-top D3 embryos refers to grade 3 embryos.

### Comparison of clinical outcomes of embryo transfer between DGC/SU and DDGC/SU groups

Given that the clinical outcomes for cleavage stage embryo transfer differ greatly from blastocyst transfer, we subdivided groups of DGC/SU and DDGC/SU into D3 embryo and blastocyte subgroups. The number of D3 embryos or blastocytes per transfer was similar in the DGC/SU and DDGC/SU groups (Table 3). The pregnancy rate, spontaneous abortion rate, implantation rate, live birth rate, birth weight and duration of pregnancy from the transfer of either D3 embryos or blastocytes were comparable in the DGC/SU group and DDGC/SU group (Table 3).

**Table 3:** Embryos transfer and clinical outcomes.

	DGC/SU	DDGC/SU	P value
ET cycles			
D3 embryos	210	147	
Blastocytes	145	120	
Number of embryos per transfer			
D3 embryos	2 [2,2]	2 [2,2]	0.8873
Blastocysts	2 [2,1]	2 [2,1]	0.6492
Pregnancy rate (%)			
D3 embryos	96/210 (45.7)	65/147 (44.2)	0.8291
Blastocytes	97/145 (66.9)	85/120 (70.8)	0.5088
Implantation rate (%)			
D3 embryos	123/390 (31.5)	83/272 (30.5)	0.7985
Blastocytes	128/230 (55.7)	111/187 (59.4)	0.4863
Spontaneous abortion rate (%)			
D3 embryos	20/96 (20.8)	19/65 (29.2)	0.2621
Blastocytes	16/97 (16.5)	12/85 (14.1)	0.6860
Live birth rate			
D3 embryos	76/210 (36.2)	46/147 (31.3)	0.3655
Blastocytes	74/145 (51.0)	71/120 (59.2)	0.2154
Birth weight (g)			
D3 embryos	2961 ± 731.5	3136 ± 673.8	0.1534
Blastocytes	2943 ± 622.6	3003 ± 689.0	0.5312
Duration of pregnancy (days)			
D3 embryos	259.3 [269.0,274.0]	262.8 [270.5,277.3]	0.0936
Blastocytes	257.0 [268.0,272.3]	259.0 [269.0,273.0]	0.5584

ET, Embryos transfer. Data are presented as the median [the first quartile, the third quartile] or count (percentage).

## Discussion

Our data showed that sperm preparation for IVF cycles by the strategy of DDGC/SU could significantly improve the sperm recovery rate from poor semen samples without adversely affecting the biological processes, including fertilization and *in vitro* and *in vivo* embryo development, suggesting that DDGC/SU could be a simple, effective and safe way to separate sperm from poor semen samples for IVF.

A previous study demonstrated that additional centrifugation time or increased centrifugation force may increase the sperm recovery rate^9^. Consistent with this finding, we found that additional centrifugation time increased the recovered sperm from both normal and poor semen samples. More importantly, additional centrifugation can separate more highly motile sperms from poor semen samples than from normal samples. Poor semen samples characterized by high viscosity, unresolved fibres or clots may result in more drag force to retard the downward movement of motile sperms during DGC. Additional centrifugation provided enough power that allowed the motile sperm thoroughly move through the entrapment. In normal semen, motile sperm can be easily separated by first DGC, and the second DGC resulted in the congregation of sperm with limited motile capacity, even immotile sperm. Therefore, a second DGC is more necessary and effective for the separation of sperm from poor semen samples.

As mentioned above, the efficiency of separating sperm from poor semen samples which are characterized by liquefaction defects, containment of too many unresolvable clots or rare sperm after one DGC, is very low. In some cases, due to a low sperm recovery rate, the enriched sperm by one DGC from poor semen samples will not meet the requirement of insemination by conventional IVF, and ICSI would be the substitute. Although the safety of ICSI is generally accepted, a recent systematic review paper showed that compared with IVF-conceived children, ICSI-conceived children may be at increased risk of autism and intellectual impairment and suggested an uncertainty of the long term safety of ICSI^19^. Furthermore, insemination by ICSI will increase the financial burden of patients. Therefore, poor semen samples processed by DDGC/SU help to reduce the total intracytoplasmic sperm injection (ICSI) cycles.

In the present study, although the DFI of fresh semen samples was not taken into consideration when deciding whether the second centrifugation should be performed, we found that the DFI was significantly higher in the DGGC/SU group than that in the DGC/SU group. Previous studies demonstrated that the level of DFI negatively correlated with semen parameters, including the sperm concentration, sperm motility, and normal sperm morphology^20–22^. Consistently, we found that the sperm concentration, percentage of PR sperm and normal sperm morphology were lower in the DDGC/SU group. In addition, the male-infertility factor ratio was significantly higher in the DDGC/SU group. The other characteristics of couples undergoing IVF cycles were comparable in the DGC/SU group and DDGC/SU group, including the age and BMI of males and females, type, duration and diagnosis of infertility, ovulation induction protocols, the total dose and duration of Gn use, endometrial thickness, average oocytes retrieved and MII oocytes. These results demonstrated that “poor” semen in the DDGC/SU group showed poor semen quality.

It has been shown that sperm with a high level of DFI negatively correlated with the outcomes of IVF-ET^18,23^. However, the findings from other studies suggested that DFI was not able to predict the outcomes of IVF^24–26^. Although no consistent conclusion was made, it is at least clear that sperm with high-level DFI will not contribute to improving the outcomes of IVF cycles. In the present study, although a higher DFI of semen was present in the DDGC/SU group, the indicators including the normal oocyte fertilization rate, top D3 embryo formation rate, blastocyte formation rate, pregnancy rate, implantation rate, live birth rate, birthweight and duration of pregnancy from either the transfer of D3 embryos or blastocytes were comparable in the DGC/SU group and DDGC/SU group. It has also been reported that sperm DFI positively correlated with **s**pontaneous abortion^27–29^. In the present study, we found that the spontaneous abortion rate following the transfer of either D3 cleavage embryos or blastocytes was comparable between the two groups. A recent study indicated that only DFI over a “threshold” may increase the risk of **s**pontaneous abortion and suggested that each laboratory may have its own threshold of DFI^27^. Therefore, we speculated that although a significantly higher DFI was observed in the DDGC/SU group, the DFI may not reach the threshold to induce spontaneous abortion. These results demonstrated that sperm from poor quality semen samples with higher DFI by DDGC/SU for IVF achieved the same outcomes as sperm from normal semen by DGC/SU, suggesting that DDGC/SU is a safe method for sperm enrichment for IVF.

Previous studies in rodents showed that increased centrifugation force would damage the cell organelles and membranes of sperm^10–12^. However, few studies have been conducted to further explore the effect of the sperm suffering excess centrifugation force on the outcomes of IVF in any species. Only one study showed that rat sperm with over centrifugation had no impact on the fertilization rate^12^. In line with that, we also found that double centrifugation did not decrease the fertilization rate, nor the other indicators mentioned above. It is known that DNA, located in the head of sperm, is highly compacted, and the most important role that sperm plays in fertilization is to transfer its DNA into oocytes. In the present study, we speculated that increased centrifugation force was not likely to damage sperm DNA, but to cause limited membrane or organelle damage that was far from affecting sperm motile capacity.

The formation of the 1PN zygote may be associated with several causes, including the fusion of male and female pronucleus, the asynchronous disappearance of male and female pronucleus, and only male or female pronucleus formation^30,31^. Theoretically, the first 2 causes will not impair further embryo development while the latter 2 will. To our surprise, we found that poor semen processed by DDGC/SU significantly decreased the ratio of 1PN zygote formation without affecting the ratio of top D3 embryo formation derived from 1PN zygotes, when compared with DGC/SU for normal semen, suggesting that poor semen processed by DDGC/SU synchronously decreased all causes-resulted the formation of 1PN.

In summary, our study verified that DDGC could effectively improve motile sperm recovery from poor semen, and showed that sperm for IVF from poor quality semen samples processed by DDGC/SU were able to achieve the same outcomes as sperm from normal semen by DGC/SU, suggesting that DDGC/SU is an effective and safe method of the treatment for poor semen samples in IVF, that is, poor semen samples can be processed by DDGC/SU for IVF in the clinic.

## Materials and Methods

### Study subjects

The data generated from a total of 529 IVF cycles (from June 2017 to June 2018) and these IVF cycle-contributed 622 transfer cycles (from June 2017 to December 2018) in Changzhou Maternal and Child Health Care Hospital, were collected and analszed in this retrospective study. Subject to semen preparation, data from IVF cycles or ET cycles were divided into two groups: the DGC/SU group and the DDGC/SU group. All of the included patients read and signed the informed consent form. This retrospective study was approved by the Ethics Committee of Changzhou Maternal and Child Health Care Hospital and Nanjing Medical University. All the treatments in the present study were performed strictly in accordance with the Declaration of Helsinki for Medical Research.

### Semen preparation

**Figure 2:**
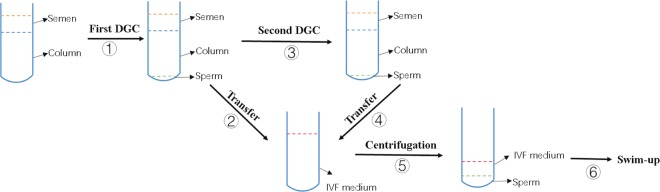
Work flow of DDGC. A total of 6 consecutive steps were involved in the entire process. Briefly, semen was placed on the density gradient column in the tube for the first DGC. After the first DGC, sperm precipitation was transferred into IVF medium. The semen in the tube on the column was subject to the second DGC. The sperm precipitation from the second DGC was also transferred to the abovementioned IVF medium. The sperm were then washed by centrifugation and re-suspended in 0.5 ml of IVF medium. Finally, sperm underwent swim-up procedure.

Patients with 2–5 days of abstinence ejaculated semen into a sterilized cup by masturbation. After liquefaction, 10 µl of semen sample was drawn on the slide and covered with a cover glass to roughly estimate sperm concentration and sperm motile parameters, including the percentage of progressive motility (PR) sperm, non-PR (NP) and immobile (IM) sperm, under a microscope with a magnification of 400X. The sperm concentration (10^6^/ml) was roughly estimated as sperm counts in a 400X visual field. The remaining sample was placed on a density gradient column consisting of 1.5 ml of lower layer and 1.5 ml of upper layer (Irvine, USA) and centrifuged at 462 g for 15 min. For DGC/SU, the sperm pellet was re-suspended in 3 ml of IVF plus medium (Vitrolife, Sweden) and washed by centrifugation at 370 g for 9 min. After that, the sperm pellet was re-suspended in 0.5 ml of IVF medium. Ten microliters of medium was used to roughly estimate PR sperm counts. Then, 0.5 ml of IVF medium was dropped gently and slowly on the surface of the sperm suspension along the inner tube wall, and the tube was placed at a vertical angle in an incubator at 37 °C and 5% CO_2_ for approximately 1.5 h before insemination for the purpose of swim up. For DDGC/SU, after the transfer of the sperm pellet obtained from the first DGC, the density gradient column was centrifuged immediately for another round. The sperm pellet from the second DGC was then mixed with the sperm pellet from the first DGC. The remaining procedures were the same as the abovementioned DGC/SU. A work flow of DDGC/SU is described in Fig. 2. For insemination, a micropipettor was used to transfer the sperm into the culture drop of the oocyte.

### Inclusive criteria of semen for DDGC/SU

In this study, semen were processed by DDGC/SU if (a) semen showed liquefaction defects, or contained too many unresolvable clots/fibres, or contained rare but high motile sperm, and (b) the sperm pellet after the first DGC was invisible or almost invisible.

### Computer-aided semen analysis system (CASA) analysis

Ten microliters of semen was added to the chamber of slides, and after a warm-up (10 min), the slide was analysed by CASA. The sperm concentration and motility-related parameters were recorded. The results of semen quality parameters presented in Table 1 and results 1, including the sperm concentration, total sperm count and ratio of PR sperm, were determined by CASA analysis.

### IVF procedures

More than half of the patients received ovarian stimulation with GnRH analogues, and the rest were treated without GnRH analogues for controlled ovarian hyperstimulation. Insemination was performed in IVF plus medium (Vitrolife, Sweden) using conventional IVF. Approximately 16–18 h after insemination, oocytes with 1 or 2 visible pronuclei were further cultured in G1 plus medium (Vitrolife, Sweden). On day 3 of culture, embryos were scored as grade I, II, III or VI based on the number, size, and shape of blastomeres and their degree of fragmentation, as previously described^32^. Grade VI embryos were discarded. D3 embryos were subjected to transfer, frozen by vitrification technology or expanded in G2-plus culture medium (Vitrolife, Sweden). Blastocysts from day 5 or 6 were scored using the system of Gardner. The usable blastocysts were frozen by vitrification technology or transferred.

### Statistical analysis

All analyses were performed by using GraphPad prism software (version 5). The chi-square test was used to compare the data of the constituent ratio. If continuous variables were normally distributed, Student’s t-test was used. If not, the Mann–Whitney U test was used. P < 0.05 was considered statistically significant.
